# Oral Lichen Planus: A Narrative Review Navigating Etiologies, Clinical Manifestations, Diagnostics, and Therapeutic Approaches

**DOI:** 10.3390/jcm13175280

**Published:** 2024-09-05

**Authors:** Houriah Yasir Nukaly, Ibrahim R. Halawani, Saja Mohammed S. Alghamdi, Araa Ghanem Alruwaili, Alhanouf Binhezaim, Rana Ali A. Algahamdi, Rayan Abdullah J. Alzahrani, Faisal Saad S. Alharamlah, Shahad Hamad S. Aldumkh, Hamad Majid A. Alasqah, Awadh Alamri, Abdulhadi Jfri

**Affiliations:** 1 Medicine Program, Batterjee Medical College, Jeddah 21442, Saudi Arabia; houriyahnukaly@gmail.com; 2 Faculty of Medicine, King Abdulaziz University, Jeddah 21589, Saudi Arabia; ibrahim1halawani@gmail.com; 3 College of Medicine, Albaha University, Albaha 65799, Saudi Arabiaranaalialgahamdi@gmail.com (R.A.A.A.);; 4 College of Medicine, Jouf University, Sakaka 72388, Saudi Arabia; araaghanem@gmail.com; 5 Saudi Board in Pediatric Dentistry [SB-PD], Department of Pedodontics, Prince Sultan Military Medical City, Riyadh 12233, Saudi Arabia; hanouf.k.hezaim@gmail.com; 6 College of Dentistry, Imam Abdulrahman Bin Faisal University, Dammam 34212, Saudi Arabia; faisal.s.alharamlah@gmail.com; 7 College of Medicine, King Saud University, Riyadh 11421, Saudi Arabia; shahadaldumkh@gmail.com; 8 College of Dentistry, King Saud University, Riyadh 11421, Saudi Arabia; ha.alasqah@gmail.com; 9 College of Medicine, King Saud Bin Abdulaziz University for Health Sciences, Jeddah 11481, Saudi Arabia; 10 King Abdulaziz Medical City, Jeddah 22384, Saudi Arabia; 11 King Abdullah International Medical Research Center, Jeddah 21423, Saudi Arabia

**Keywords:** oral lichen planus, chronic inflammation, oral lichenoid keratosis, autoimmune, corticosteroids

## Abstract

**Background/Objectives**: Oral Lichen Planus (OLP) is a common immune-mediated inflammatory disorder affecting the oral mucosa, impacting 0.5% to 2% of the global population, primarily middle-aged women. Immunological dysregulation is a key factor in OLP’s pathogenesis, involving CD4+ T helper and CD8+ T cytotoxic cells. The World Health Organization (WHO) classifies OLP as a potentially malignant disorder, with a risk of oral squamous cell carcinoma (OSCC) developing in up to 2% of lesions. This narrative review aims to provide a comprehensive overview of the etiopathogenesis, clinical manifestations, diagnostic criteria, and therapeutic strategies for OLP, informing clinical practice and guiding future research. **Methods**: A review of the literature from the PubMed and Google Scholar databases was conducted up to December 2023, focusing on studies addressing the etiopathogenesis, diagnosis, clinical manifestations, and treatment of OLP. **Results**: OLP’s pathogenesis is driven by immune dysregulation, with CD4+ and CD8+ cells playing crucial roles. Clinically, OLP presents as reticular, erosive, bullous, and plaque-like lesions. Diagnosis relies on clinical examination, histopathology, and direct immunofluorescence. Recent advancements in diagnostic markers and imaging techniques have improved detection and monitoring. Treatment primarily involves corticosteroids, but novel therapies such as curcumin, retinoids, and laser therapy are increasingly used for their effectiveness and reduced side effects. These treatments show promise in symptom reduction and recurrence prevention, although long-term data are needed. **Conclusions**: Regular screenings and biopsies are essential due to OLP’s likelihood of malignant transformation. This study urges further investigation into long-term results, improved diagnostic techniques, and evidence-based treatment regimens.

## 1. Introduction

Lichen planus, an immune-mediated inflammatory condition, primarily involves the mucous membranes of the oral cavity and the skin. It is characterized not only by its oral manifestations but also by its potential to affect the cutaneous surfaces, presenting with distinctive lesions [1]. Its recognition dates back to as early as 1866, and the term “lichen planus” (LP) was introduced by Wilson [2]. OLP ranks as one of the most common dermatological conditions in the oral cavity, impacting around 0.5% to 2% worldwide [3]. A preference for females at a ratio of 2:1 is observed, and the condition typically begins most frequently during middle age [1]. Although rare, OLP can also occur in children [4,5,6]. OLP lesions can manifest in various presentations, including reticular and papular, plaque-like, atrophic, erosive, and bullous forms which present clinically with oral discomfort or pain while eating [7,8,9]. While its exact cause remains unexplained, several theories suggest that immune dysregulation may initiate the persistent inflammatory process in OLP [10,11]. CD4+ T helper (Th) cells and CD8+ T cytotoxic cells play significant roles in the development of OLP [10,12]. The WHO has categorized OLP as a potentially malignant disorder, with its most serious complication being OSCC [13]. The malignant transformation risk of OLP to OSCC is 1% to 2% of cases, with the erosive subtype being the highest risk [14,15]. The WHO has suggested that being female, having the erosive type, and its location on the tongue are factors that increase the risk of OLP malignancy transformation [16,17]. 

The primary aim of this review is to provide a comprehensive exploration of the latest insights into the factors contributing to the development of OLP, the diagnostic methods and techniques, and the various strategies for effectively managing this complex oral disorder. Many studies have focused on OLP with different views; in this study, we tried to present an overview of recent perspectives on etiologies, diagnosis, and management. Our review seeks to consolidate and present a comprehensive body of information that will be a valuable resource for healthcare professionals, researchers, and individuals seeking a more profound comprehension of this condition.

## 2. Materials and Methods

A comprehensive literature search was performed on PubMed and Google Scholar using the search terms “Oral Lichen Planus Pathogenesis”, “Oral Lichen Planus Diagnosis”, and “Oral Lichen Planus Management”. Studies published up to December 2023 were considered for review. Reference lists of included records were screened for additional studies of interest. Full-text articles deemed relevant based on title and abstract screening were obtained and evaluated for inclusion and exclusion criteria. Two authors (HYN and IRH) independently screened titles, abstracts, and full texts for inclusion. Any discrepancies in study selection were resolved by consensus with a third independent author (AJ). All studies in English with a primary focus on the etiopathogenesis, clinical manifestations, diagnostic criteria, and treatment strategies of OLP were evaluated. Inclusion was restricted to studies that provided detailed information on OLP, including conventional and historical diagnostic approaches, recent advancements in diagnostic techniques, and therapeutic strategies. Studies that did not meet these criteria were excluded from the review.

**Figure 1:**
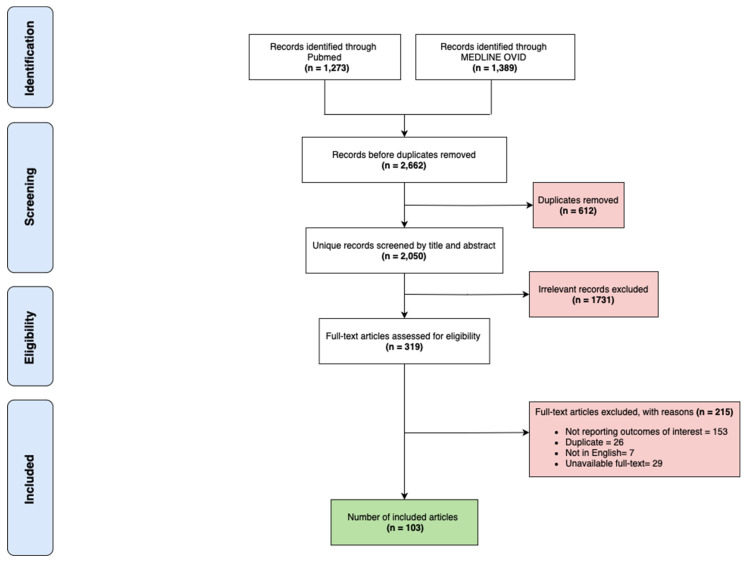
Flowchart detailing the full process of study inclusion.

Inclusion Criteria: (1) Studies in English with a primary description of the etiopathogenesis, clinical manifestations, diagnosis, and treatment of OLP. (2) Articles covering various aspects of OLP, including its conventional and historical diagnostic approaches and management strategies. (3) Recent advancements in diagnostic techniques and therapeutic approaches. Exclusion Criteria: (1) Studies describing conditions other than OLP. (2) Articles not providing detailed information relevant to the study aims. (3) Non-English language studies. Figure 1 details the full process of study inclusion.

## 3. Results

### 3.1. Etiology of Oral Lichen Planus

OLP is a chronic inflammatory condition affecting the oral mucosa; however, its etiology remains unclear. Despite extensive studies, the definitive cause remains elusive, attributing OLP’s pathogenesis to multifactorial origins and interweaving genetic, immunological, and environmental components [18].

#### 3.1.1. Immunological Links

According to the prevailing theory, oral lesions result from an aberrant immune response mediated by T cells, where autoreactive T cells trigger apoptosis of the epithelial cells of the mouth [18]. As a known aggravator of immune-mediated conditions, stress is recurrently linked to exacerbations of OLP, providing insight into possible psycho-neuroimmunological mechanisms [18,19].

#### 3.1.2. Genetic Predisposition

Recent research suggests a potential genetic predisposition to OLP, as variations in genes associated with the immune response, such as HLA antigens, are observed in numerous OLP patients [20,21,22,23,24]. These genetic susceptibilities could potentiate abnormal immune reactions within the oral mucosa, culminating in clinical manifestations of OLP [20]. A case series of eight Chinese families corroborates the supposition that genetic factors may play a significant role in the origin of OLP [20]. There are observed associations between genetic polymorphisms in the Vitamin D receptor (VDR) and susceptibility to OLP in the Han Chinese population [21]. The origins and developmental mechanisms of OLP remain largely indeterminate, but there are indications that genetic predisposition plays a significant role in the disease’s onset [22]. Triggers that may activate autoimmune reactions and lead to the manifestation of OLP include genetic predispositions, dermal and mucosal microbiota, environmental aspects, and epigenetic alterations [23]. Nonetheless, comprehensive studies are imperative to gain a profound understanding of OLP’s genetic underpinnings.

#### 3.1.3. Environmental Triggers

OLP is acknowledged as a multifaceted ailment influenced by a spectrum of triggering and aggravating agents which encompass psychological stress, pharmaceuticals, dental materials such as gold or amalgam, and viral infections such as Epstein–Barr, varicella zoster, and Hepatitis C viruses along with environmental factors [24]. Moreover, compounds resulting from peroxidation, antioxidants, cortisol, and immunoglobulins are potential biomarkers for predicting the onset of OLP [25]. The initiators of autoimmune responses, dictating the manifestation of OLP, include genetic predisposition, dermal and mucosal microbiota, environmental influences, and epigenetic alterations [26,27]. The convergence of these elements may engender an aberrant immune reaction, culminating in the distinctive lesions observed in OLP [24,25].

#### 3.1.4. Microbial Associations

Recent investigations in microbiology have revealed connections between shifts in the oral microbiota and the onset of OLP, indicating a potential interrelationship between microbial disbalance and immune dysregulation [28,29,30]. Imbalances in the gut microbiota precipitate a breakdown in the structural integrity of the intestinal barrier, resulting in increased gut permeability and migration of bacteria and their by-products into the bloodstream. This process may serve as a plausible mechanism for T-cell activation, potentially culminating in the onset of OLP [29,30]. Current research in oral microbiota functionality and host-microbiota interactions needs to be more comprehensive, warranting further investigation [28]. While studies have scrutinized the microbiome profiles within both lesions and adjacent healthy mucosal regions among OLP patients, suggesting microbial dysbiosis as a potential player in OLP pathogenesis, the precise involvement of oral microbiota in OLP development remains elusive [30]. Investigations have been conducted into salivary mycobiome dysbiosis and its potential ramifications for shifts in bacteriome composition and host immunity in OLP [28,31]. However, a comprehensive understanding of the intricate interplay between microbial imbalance and immune dysregulation in OLP necessitates additional research [28].

#### 3.1.5. Hormonal Influence

Several studies have established a relationship between hormonal fluctuations and the development of OLP, particularly in postmenopausal females. The transition through menopause causes significant endocrine shifts in women, notably affecting the production of sex steroid hormones [32]. Elevated levels of circulating estrogen in individuals with OLP have been linked to increased severity of lesions. Nevertheless, the precise contribution of hormonal fluctuations to the genesis of OLP remains uncertain, necessitating additional research to comprehensively elucidate the intricate interplay between hormonal influences and OLP pathophysiology [32,33,34,35].

#### 3.1.6. Medications Associated with Drug-Induced Lichenoid Reactions in Oral Lichen Planus

OLP lesions can also be triggered or exacerbated by certain medications, leading to drug-induced lichenoid reactions. Drugs commonly associated with these reactions include antihypertensives such as beta-blockers and ACE inhibitors, nonsteroidal anti-inflammatory drugs (NSAIDs), antimalarials such as hydroxychloroquine, and certain classes of antibiotics. Other medications that have been implicated include thiazide diuretics, certain antidiabetic drugs, and some psychiatric medications. The mechanism behind these reactions often involves an immune-mediated response, where the drug or its metabolites are thought to act as haptens, leading to a T-cell-mediated inflammatory reaction. Identifying and discontinuing the offending drug can lead to improvement or resolution of the lesions [36].

The pathogenesis of OLP is a multifaceted process that reflects the complex interplay between genetic, immunological, environmental, microbial, hormonal, and medication-related factors. Genetic predispositions, such as variations in immune response genes, may prime individuals for an exaggerated immune response, particularly involving autoreactive T cells that target epithelial cells. Environmental triggers, including stress, pharmaceuticals, and dental materials, can further modulate this immune response, often acting as haptens or stressors that exacerbate the inflammatory cascade. Additionally, microbial dysbiosis in the oral and gut microbiota may disrupt immune homeostasis, contributing to the activation of immune cells and the perpetuation of inflammation. Hormonal fluctuations, especially in postmenopausal women, may influence the severity and progression of OLP through their effects on immune regulation and epithelial cell turnover. Finally, certain medications can induce lichenoid reactions, mimicking OLP by provoking a T-cell-mediated immune response. Together, these interconnected factors highlight the complexity of OLP pathogenesis, where each element may contribute to the onset and persistence of this chronic inflammatory condition.

### 3.2. Clinical Presentation

**Figure 2:**
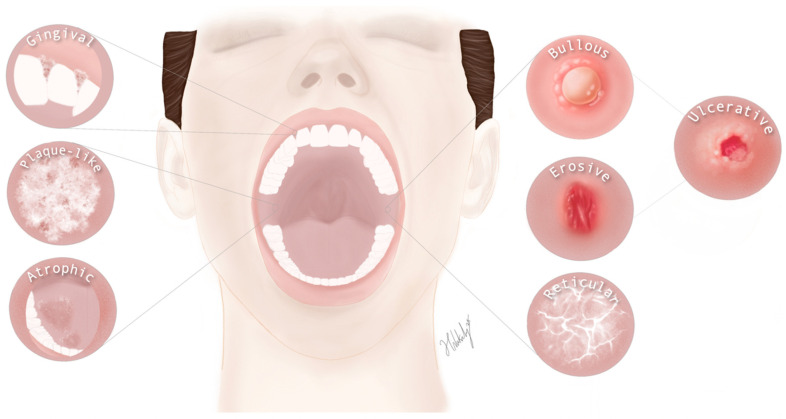
Illustration of the different forms of OLP, including gingival involvement. Insets highlight characteristic manifestations such as reticular, erosive, plaque-like, atrophic, and bullous OLP, demonstrating the various appearances it can exhibit.

OLP presents a wide range of clinical manifestations, and its diagnosis often relies on a combination of clinical findings, histopathological examination, and other additional tests [6]. A defining feature of OLP is Wickham’s striae, named after the dermatologist Sir William Wickham. These hallmark striae manifest fine, white, or off-white lines or streaks that appear in a web-like pattern on the mucosal surfaces affected by OLP, most commonly on the buccal mucosa, tongue, and gingiva. The exact etiology remains under investigation, with theories proposing increased epithelial keratinization, subtle inflammatory changes, or accentuated surface ridges due to epithelial thinning as potential explanations. It is important to consider that lichenoid lesions can also appear as Wickham’s striae, necessitating a differential diagnosis to confirm lichen planus. We herein outline the various forms of OLP as illustrated in Figure 2.

#### 3.2.1. Reticular OLP

Reticular OLP is the most common clinical subtype characterized by delicate white, lacy lines or papules on the oral mucosa, typically appearing bilaterally on the buccal mucosa, tongue, and gingiva [37]. This distinctive reticular pattern, resembling a web-like network, is a hallmark diagnostic feature of OLP, aiding in its identification. Despite its relatively benign appearance, reticular OLP can sometimes cause mild discomfort or a burning sensation [38]. It is imperative to emphasize that OLP can coexist with cutaneous disease. A comprehensive and thorough extra-oral examination is essential for providing appropriate management [39].

#### 3.2.2. Erosive OLP

Erosive oral lichen planus (EOLP) is a distressing variant of this chronic inflammatory condition, featuring painful ulcers, erosions, and erythematous areas within the oral cavity [40]. These lesions commonly occur on the buccal mucosa, tongue, gums, and other oral surfaces, are often described as “punched out” or “crater-like”, and are occasionally accompanied by Wickham’s striae and white lines [41]. EOLP significantly impairs daily activities such as eating and speaking, causing discomfort, a burning sensation, and heightened sensitivity in affected areas. Early diagnosis and tailored intervention are vital for symptom relief to improve the quality of life of patients and prevent ongoing chronic inflammation that could contribute to the risk of SCC transformation. EOLP can be chronic, with recurrent episodes, but some individuals may experience extended periods of remission [41,42].

#### 3.2.3. Bullous OLP

Bullous OLP is a distinctive but rare clinical presentation affecting oral mucosa [6]. Individuals with OLP develop fluid-filled vesicles or bullae within the oral cavity, which can be painful and uncomfortable. These blisters are commonly found on the buccal mucosa, tongue, and gums and may rupture easily, resulting in painful ulcers [43]. While bullous OLP is less prevalent than other OLP subtypes, its unique characteristics and potential for symptom exacerbation underscore the importance of accurate diagnosis and tailored management. The treatment aims to alleviate discomfort, promote lesion healing, and prevent recurrences, ultimately improving oral health and quality of life [44]. It can be a chronic condition with intermittent flare-ups and remission periods requiring close monitoring [6,44].

Additionally, ulcerative OLP arises as a secondary condition to bullous and erosive types, leading to painful ulcers within the oral cavity [45]. These ulcers can also be single or multiple and are found predominantly on the buccal mucosa, tongue, and gums, although they can occur on the lips, palate, and floor of the mouth. The ulcers may be red or white and can sometimes be surrounded by white lines known as Wickham’s striae, although their absence does not exclude the diagnosis of ulcerative OLP [25,46]. Ulcerative OLP is particularly distressing due to its severity. It can cause significant discomfort and pain which can hinder eating, speaking, and drinking, profoundly impacting the patient’s quality of life. Early diagnosis and proactive management are essential to alleviating symptoms, promoting healing, and preventing complications including scarring or the development of oral cancer [9,45].

#### 3.2.4. Atrophic OLP

Atrophic lichen planus affects both the skin and mucous membranes. In the oral cavity, it is referred to as atrophic OLP. This variant is characterized by areas of mucosal thinning, resulting in a smooth, shiny appearance, often observed on the tongue and occasionally in other oral locations [46]. While atrophic OLP may not cause the pronounced pain seen in erosive forms, it can still cause patients distress due to its aesthetic changes and potential mucosal sensitivity [47]. In general, atrophic lichen planus, whether on the skin or mucous membranes, is marked by thin, smooth patches that may be red or purplish. These lesions can appear on various body parts, often accompanied by Wickham’s striae. They can be asymptomatic or cause burning or itching sensations. While the condition is chronic, periods of remission may occur [46,48].

#### 3.2.5. Plaque-like OLP

Individuals with OLP and plaque-like OLP exhibit flat white patches on the oral lining, which may appear as single or multiple lesions of varying sizes and shapes [49]. This type is more prevalent among smokers. These patches are most frequently found on the buccal mucosa, tongue, and gums but can also occur on the lips, palate, and floor of the mouth. The lesions can be smooth or slightly raised and may be surrounded by Wickham’s striae, which indicate inflammation [50,51]. This type can be asymptomatic or cause a burning or itching sensation. The lesions may become irritated, occasionally leading to scaling or crusting [51].

#### 3.2.6. Gingival Involvement

Gingival involvement in OLP is very common and can manifest in various ways. The primary presentation is often atrophic gingivitis, which manifests as bright red lesions due to epithelial thinning, exposing the underlying vasculature. The surface is typically smooth and lacks healthy gingival texture, sometimes appearing shiny. These lesions primarily affect the attached gingiva and interdental papillae, varying in size and shape. Borders can be well-defined or blurred, with Wickham’s striae appearing less frequently. Patients may experience burning, soreness, or pain, particularly with extensive lesions. Importantly, while stable, atrophic gingivitis can progress to erosive lichen planus with deeper ulcerations [52]. 

Additionally, gingival OLP may appear as:

1. **Plaque-like Lesions:** These white or grayish lesions on the gingiva may be smooth or slightly raised, sometimes encircled by Wickham’s striae.
2. **Ulcerative Lesions:** Painful red or white ulcers can develop on the gingiva, either as single or multiple lesions, with varying depths.
3. **Bullous Lesions:** Though rare, gingival OLP can lead to blister formation on the gums which may easily rupture, causing painful ulcers [53,54].
4. **Desquamative Lesions**: Desquamative gingivitis, a hallmark presentation in OLP, manifests with erythematous and erosive gingival alterations, resulting in a desquamated appearance. It is characterized by erythema and occasional vesicle formation on the gingiva. While OLP is an autoimmune condition, plaque and bacterial build-up can exacerbate inflammation and potentially worsen desquamative gingivitis [55].

Symptoms of gingival OLP encompass gingival bleeding, difficulties in chewing and swallowing, and gum pain. The chronicity of gingival OLP is marked by fluctuating symptom intensity, although extended remission phases are observed in certain cases [52,54].

#### 3.2.7. Associated Symptoms

OLP may be accompanied by a range of associated symptoms that can vary in intensity among affected individuals. Commonly reported associated symptoms of OLP include:

**Pain:** Many individuals with OLP experience pain or discomfort in the affected oral areas, ranging from mild soreness to severe burning or stinging sensations, particularly in erosive and ulcerative forms of OLP [6,56].

**Burning Sensation:** A persistent burning or tingling sensation, often described as a “burning mouth” or “burning tongue”, is frequently reported and can worsen with eating certain foods or poor oral hygiene practices [44]. It is especially felt with spicy, acidic, or abrasive foods, potentially resulting in dietary restrictions and weight loss in severe cases [9].

**Dry Mouth [Xerostomia]: The presence of** OLP lesions can result in reduced saliva production and cause dryness in the mouth, which can contribute to oral discomfort, difficulty in swallowing, halitosis, and an increased risk of dental caries [56].

**Altered Taste [Dysgeusia]:** Some OLP patients report changes in taste perception, often describing a metallic or bitter taste in their mouths that affects their enjoyment of food [54,56].

**Speech Difficulties:** Extensive OLP lesions, or those affecting critical speech areas such as the tongue, may lead to difficulties in articulation and pronunciation [51].

**Psychological Impact:** Chronic oral discomfort has a significant impact on quality of life; it affects daily activities such as eating and speaking and can have psychological consequences, potentially causing anxiety, depression, or decreased quality of life [6,56]. 

### 3.3. Diagnosis 

Diagnosis of OLP is challenging because of overlapping clinical and histopathological features. Investigations include biopsy (histopathological and immunofluorescence), in addition to other tests [57,58,59].

#### 3.3.1. Mucosal Biopsy

Lichen planus is typically diagnosed based on clinical presentation in classic cases; however, histopathological confirmation via biopsy is recommended, particularly for atypical presentations. A 4 mm punch biopsy should be adequate for both cutaneous and oral assessments [59]. Histopathologic examination typically reveals a characteristic “saw-tooth” pattern of epidermal hyperplasia, hyperparakeratosis with thickening of the granular cell layer, and vacuolar alteration of the basal layer of the epidermis with an intense infiltration [mainly T cells] at the dermal–epidermal junction. A 4 mm punch biopsy of perilesional skin for direct immunofluorescence may be added to the workup when bullous lesions, pemphigus, or bullous pemphigoid are present. Moreover, in the histopathological evaluation of lichenoid lesions, the presence of eosinophils is particularly significant. While eosinophils are typically absent or minimal in classical OLP, their presence can indicate other lichenoid disorders such as drug-induced reactions or hypersensitivity conditions. Accurately identifying the underlying diagnosis is essential, as it significantly improves the prognosis and guides critical management strategies [60,61].

#### 3.3.2. Other Tests

Hematological assessments play a pivotal role in the comprehensive evaluation of OLP. It may be performed in some cases to rule out underlying systemic conditions or to monitor the general health of the patient. Some common hematological investigations that may be considered in the context of OLP include Complete Blood Count [CBC], Erythrocyte sedimentation rate, and Hb C Ag [hepatitis C virus]. A patch test is recommended to rule out possible allergies, especially to substances used in dental materials.

**Final diagnosis of OLP based on modified WHO diagnostic criteria (Table 1):**

To establish a definitive diagnosis, the integration of both clinical observations and histopathological findings is essential:

- OLP—A diagnosis of OLP requires fulfillment of both clinical and histopathological criteria.
- OLL—The term OLL will be used under the following conditions.
  - Clinically typical of OLP but histopathologically only “compatible with” OLP.
  - Histopathologically typical of OLP but clinically only “compatible with” OLP.
  - Clinically “compatible with” OLP and histopathologically “compatible with” OLP [62].

Finally, Table 2 provides a comprehensive summary of the differentiation between OLP and OLL [63].

### 3.4. Malignant Potential

OLP is a condition that is considered potentially malignant as it can develop into OSCC [66]. The progression of OLP to OSCC is a focal point of research due to the implications of genetic and cellular changes in this progression. Among the notable findings, a reduction in succinate dehydrogenase enzyme B (SDHB) has been associated with the development of OSCC, suggesting a critical role for SDHB in the malignant transformation of OLP [67]. Furthermore, the DNA damage caused by inducible nitric oxide synthase (iNOS) in OLP can lead to the accumulation and mutation of the p53 protein, which is observed in both OLP and OSCC [68].

Apart from genetic and epigenetic alterations, cellular changes also play a role in the progression of OLP to OSCC. For instance, cytokines and chemokines released by infiltrating T cells in OLP could cause modifications in the proteins of cells, potentially contributing to the advancement of OSCC [69]. This is supported by evidence that observed expression during the transformation from OLP to indolent OLP with enrichment of pathways related to actin cytoskeleton, mitochondrial dysfunction, and oxidative phosphorylation. This finding highlights the interplay between the immune system, inflammation, and the progression towards oral cancer [70].

However, it is essential to note that the progression of OLP to malignancy can differ among different population groups. Studies report that a small percentage, around 0.5%, of 327 patients with OLP develop cancer within a seven-year timeframe. While this might seem like a low number, it is still significantly higher compared to the risk faced by the general population. This finding highlights the increased vulnerability experienced by individuals diagnosed with OLP [71,72].

The picture regarding OLP transformation to OSCC is further complicated by the variability in reported risk. Studies have documented a wide range, from a low of 0.06% to a high of 6.5% [71,72]. Interestingly, some research even suggests no increased risk compared to the general population. This inconsistency underscores the need for further investigation into the factors that influence the potential for OLP to become cancerous [73,74].

The prevalence of OLP in the south Indian population is 2.6%, with transformation rates to malignancy ranging from 0.5% to 2%, highlighting significant variability in both prevalence and transformation rates across different populations [75]. Intensive research efforts have focused on exploring factors influencing OLP’s progression to malignancy. Age and gender have emerged as potential risk factors, yet the evidence remains inconclusive. For instance, some studies have found no direct correlation between age and increased risk of transformation, while others have identified a gender disparity, indicating a higher risk among women aged 60 to 70. Lifestyle choices, particularly tobacco and alcohol consumption, have also been investigated for their potential to exacerbate the risk of OLP transforming into malignancy [76,77]. In addition, the anatomical location of OLP lesions, especially those on the tongue, alongside the presence of longstanding, erosive, and atrophic lesions, have been associated with an elevated risk of malignancy [78,79,80].

It is worth noting that when determining the risk of OLP transforming into malignancy, the assessment of dysplasia is considered the most reliable method. However, this approach does have its limitations, such as variations between intraobservers and interobservers. Researchers have started exploring biomarkers as an alternative to address these challenges. For instance, in OLP, Podoplanin and ABCG2 have shown potential as markers for assessing the risk of transformation. This offers an avenue to assess risk while reducing reliance solely on evaluating morphological changes [73]. Other markers for the early detection of the malignant transformation of OLP into OSCC include Intercellular Adhesion Proteins, Cell Cycle Regulators, Apoptotic Biomarkers, which include proteins such as p53, p63, p73, and others, Tissue Remodeling Factors, enzymes such as matrix metalloproteinases (MMPs) and cathepsin B (CB), Inflammatory Mediators, Growth Factor Receptors, and Hormonal Receptors. Each of these markers provides a potential target for early diagnosis, prognostic evaluations, and therapeutic interventions, highlighting the complex interplay of molecular pathways in the malignant transformation of OLP [81]. 

Additionally, smoking has been identified as a significant risk factor. Smokers with OLP show a higher rate of malignant transformation compared to non-smokers, likely due to the synergistic effects of carcinogenic compounds in tobacco on already compromised oral mucosa. In addition, some studies suggest that OLLs, particularly those that persist or are left untreated, may also harbor a risk for malignant transformation, although this risk is generally perceived to be lower than that of classic OLP [82].

Furthermore, the long-term outcomes for patients with OLP indicate a notable risk of malignant transformation to OSCC. A 33-year cohort study conducted in northern Italy, which included 3173 patients with histopathologically confirmed OLP, reported a malignant transformation rate of 2.58%, with a median time of approximately 96 months from initial diagnosis to the development of OSCC. Factors that significantly increased the risk of malignancy included older age, the presence of the red form of OLP, and a limited number of involved sites. These findings underscore the importance of regular, long-term follow-up for OLP patients, particularly those with identified risk factors, to facilitate early detection and intervention for potential malignant transformation. Regular monitoring, ideally on an annual basis, by trained clinicians is recommended to optimize patient outcomes and manage the risk of cancer development effectively [83].

### 3.5. Treatment

#### 3.5.1. Corticosteroids

The primary approach to treating lichen planus, as recommended by the European Academy of Dermatology and Venereology (EADV), involves the use of corticosteroids, which have been shown to effectively reduce symptoms promptly. These medications are favored for their anti-inflammatory properties, with topical corticosteroids such as triamcinolone acetonide, fluocinonide, and clobetasol propionate, including clobetasol propionate 0.5% applied twice daily for two weeks, being successfully utilized in the treatment of OLP. Although effective, extended use of topical corticosteroids can lead to side effects, including thinning of the mucosal lining and an increased risk of candidiasis. In an innovative approach, Sridharan and Sivaramakrishnan (2021) have suggested that combining corticosteroids with vitamin D or ozonated water could enhance treatment outcomes [84,85]. In addition to topical corticosteroids, intralesional steroids such as Triamcinolone Acetonide have emerged as a complementary approach in the management of OLP. Intralesional corticosteroids present several potential advantages over topical treatments for OLP, including a higher concentration of the drug directly at the lesion site and fewer systemic side effects. They are especially effective for treating erosive or ulcerative forms of OLP, providing quick therapeutic relief. However, the use of intralesional injections has been limited due to the pain and discomfort experienced at the injection site, as well as the risk of tissue atrophy [86]. Despite these benefits, further clinical trials are necessary to conclusively determine their efficacy and establish optimal dosing regimens in comparison to other treatment modalities for OLP. 

#### 3.5.2. Curcumin

Researchers continue exploring treatments for OLP, and one up-and-coming candidate is curcumin—an extract from turmeric known for its anti-inflammatory properties. A systematic review demonstrated that curcumin effectively improves outcomes and reduces pain in patients with OLP [85]. Curcumin is administered at a dose of 300–600 mg orally thrice daily for 8–12 weeks, with evaluations for efficacy and gastrointestinal tolerance.

#### 3.5.3. Systemic Therapies 

Systemic therapies are used in instances when topical treatments prove ineffective, with an initial recommended dosage of 0.5–1 mg/kg/day for severe cases. This should be tapered over 3–4 weeks to prevent systemic effects including immunosuppression and osteoporosis. These therapies can include oral corticosteroids, retinoids, and other medications that suppress the immune system. While corticosteroids are commonly prescribed for their anti-inflammatory properties, they can have side effects including weakening the immune system, disrupting sleep patterns, and causing bone demineralization [85]. Monitoring usage and carefully weighing the potential risks against the potential benefits is essential.

#### 3.5.4. Immunosuppressants

Tacrolimus has shown promise in reducing symptoms of OLP [84]. Furthermore, dapsone is also used to treat OLP. They help regulate the immune response and reduce inflammation. A study found that dapsone, tacrolimus, and topical retinoids were as effective as steroidal drugs but had fewer side effects, thus offering an alternative treatment option [87]. Tacrolimus ointment [0.1%] is applied twice daily to the affected areas for up to 4 weeks, with close monitoring for irritation and systemic absorption. Meanwhile, Dapsone is prescribed at doses of 50–150 mg/day for up to 6 months, and retinoids at 0.5 mg/kg/day for up to 16 weeks, with vigilant monitoring for potential side effects associated with each medication.

Other immunosuppressant medications such as cyclosporine, azathioprine, or topical immunosuppressants such as tacrolimus and pimecrolimus have been employed in managing OLP. Despite their proven effectiveness, it is essential to consider side effects such as an unpleasant taste during use, initial burning sensations upon application, potential nephrotoxicity (hypertension), and associated costs [86].

#### 3.5.5. Retinoids

Both topical and systemic retinoids have been used to treat OLP. Topical retinoids such as tretinoin, isotretinoin, and fenretinide (0.1% gel) have been found to reduce the presence of plaque lesions and decrease the chances of recurrence after treatment. Systemic retinoids such as etretinate, isotretinoin, and tretinoin have also been used, but their usage is limited due to side effects such as cheilitis, liver damage, and teratogenic effects. Notably, Temaroten has effectively treated OLP with fewer side effects [86].

#### 3.5.6. Laser Therapy

Laser therapy, particularly the use of a CO_2_ laser, has shown potential in adjunctive therapies, with several studies supporting its effectiveness [88]. A systematic review highlighted its utility, especially in managing the erosive and atrophic variants of OLP [87]. The CO_2_ laser treatment is tailored based on lesion severity and extent, typically conducted in a single session with the possibility of follow-up treatments depending on patient response. It focuses on targeting inflammation using lasers, showing promise in managing OLP [18]. Notably, the Nd; YAG laser has explicitly improved symptoms and the clinical score of lichen planus lesions; it is considered a safe and effective treatment option [89,90]. Additionally, low-level laser therapy has proven effective in treating adult patients with lichen planus [91]. 

#### 3.5.7. Photodynamic Therapy (PDT)

Photodynamic therapy (PDT) is another treatment option. It works by using a photosensitizing agent and light to eliminate abnormal cells. The sessions are conducted weekly for 4–6 weeks with close observation for phototoxic reactions. Studies have shown that PDT effectively reduces the pain and inflammation associated with OLP [18]. A clinical study confirmed that PDT mediated by methylene blue significantly alleviated the signs and symptoms of OLP without any adverse effects [92]. This indicates that PDT holds promise as a non-invasive alternative for managing OLP and highlights the potential of narrowband ultraviolet B (NB-UVB) phototherapy as an effective and safe option, especially when traditional therapeutic strategies falter [93,94].

#### 3.5.8. Vitamin D

Vitamin D has been increasingly recognized for its potential therapeutic benefits in managing OLP, largely attributable to its immunomodulatory and anti-inflammatory properties. These characteristics suggest a vital role for vitamin D in the pathophysiology of OLP. A systematic review highlighted that supplementation with vitamin D, alongside conventional steroid therapies, significantly ameliorated symptoms of OLP compared to placebos or steroids alone. This effect is likely mediated through vitamin D’s capacity to regulate keratinocyte proliferation and differentiation, crucial processes in the mucosal response characteristic of OLP. Interestingly, Sridharan and Sivaramakrishnan (2021) suggested that combining corticosteroids with vitamin D or ozonated water could also improve treatment results [84]. Despite promising preliminary findings, further research involving larger cohorts is essential to conclusively delineate the role of vitamin D in OLP and establish recommended dosing regimens. The existing evidence, however, positions vitamin D as a potentially valuable adjunctive treatment in OLP management strategies [95].

#### 3.5.9. Biologics 

In the treatment of refractory OLP, various biologic therapies targeting cytokines such as anti-TNF-alpha, anti-IL17, and anti-IL12/23 have been employed with variable outcomes. TNF-alpha inhibitors such as etanercept, infliximab, and adalimumab have shown promise, though there are reports of these agents exacerbating OLP. Additionally, therapies such as Alefacept and agents targeting IL-17 and related pathways (e.g., ustekinumab, guselkumab, secukinumab, and tildrakizumab) have demonstrated efficacy, particularly in reducing the Th1 and Th17/Tc17 cellular mucosal infiltrate, suggesting a key role for IL-17-producing T cells in OLP pathogenesis. Conversely, rituximab has produced inconsistent results, emphasizing the need for further research to optimize treatment strategies for OLP [8]. 

#### 3.5.10. Janus Kinase Inhibitors

Currently, the literature lacks strong evidence supporting the use of JAK inhibitors in the treatment of OLP. The available data are primarily limited to isolated case reports. However, a small number of these reports have indicated the potential efficacy of specific JAK inhibitors, such as baricitinib and upadacitinib, in managing OLP symptoms. These preliminary findings suggest a possible therapeutic role for JAK inhibitors, warranting further investigation through more extensive studies to validate their effectiveness and safety in OLP treatment [8].

#### 3.5.11. Platelet-Rich Plasma

Zhang et al. (2022) highlighted the promising advancements in managing OLP with autologous blood platelets, particularly using Platelet Concentrates (PCs) such as Platelet-Rich Plasma (PRP) and injectable Platelet-Rich Fibrin (i-PRF). Their systematic review found that PCs are effective in reducing pain and symptoms associated with OLP, delivering high concentrations of growth factors and cytokines to promote tissue repair and reduce inflammation. Unlike corticosteroids, which are associated with adverse effects such as mucosal atrophy and candidal infections, PCs offer a safer alternative with fewer side effects. Although the initial response to PRP may be slower compared to traditional corticosteroid treatments, the long-term outcomes are comparable or even superior, particularly in patients unresponsive to conventional therapies. Similarly, Sriram et al. (2023) noted the anti-inflammatory, antioxidant, and immunomodulatory properties of PRP, which not only alleviates pain and lesion severity but also reduces recurrence rates compared to corticosteroids. Their findings suggest that PRP could be especially beneficial for patients who experience significant side effects from other treatments. However, both studies emphasize the need for further research with larger sample sizes to establish standardized protocols, optimal dosages, and the long-term safety of PCs in the management of OLP [96,97]. 

Lastly, Injectable Platelet-Rich Fibrin (i-PRF) has emerged as a promising therapeutic option for the management of OLP. A meta-analysis by Gupta et al. highlighted the potential of i-PRF in reducing the severity of OLP symptoms, including pain and lesion size. The studies included in the review demonstrated that i-PRF contributes to tissue regeneration and wound healing, making it a promising alternative to traditional treatments such as corticosteroids. Despite the variability in study designs and small sample sizes, i-PRF was shown to effectively reduce pain and improve lesion size, with outcomes comparable to those achieved with standard treatments such as triamcinolone acetonide and methylprednisolone acetate. Additionally, i-PRF showed an increase in patient satisfaction, possibly due to its regenerative properties and minimal adverse effects. These findings suggest that i-PRF could be an effective adjunctive treatment for OLP, offering benefits in terms of pain management and lesion healing while avoiding the adverse effects associated with long-term corticosteroid use. However, further research with larger sample sizes and standardized protocols is needed to confirm these findings and establish i-PRF as a mainstay treatment for OLP [98].

#### 3.5.12. Other Treatment Options

Other interventions, including surgical excision and cryosurgery, have shown potential in treating erosive OLP that does not respond well to other therapies, but these procedures have drawbacks. Cryosurgery, for example, may lead to relapse, with more intense symptoms often accompanying lesion formation during the healing process. Additionally, other surgical strategies, such as soft tissue grafts and free gingival tissue grafts, have been utilized to treat localized OLP lesions, further expanding the available treatment options [85]. Lifestyle choices and dietary habits also influence the course of OLP [88]. Avoiding known triggers such as spicy foods, inadvertent dental injuries, and psychological stress can help symptom mitigation [1]. Moreover, recent research suggests that incorporating a diet rich in antioxidants could potentially deter OLP’s progression, offering a novel perspective on its management [99].

Local anesthetics and analgesics have proven beneficial for immediate symptomatic relief [100]. Preparations such as Benzydamine mouthwash and lidocaine gel are frequently prescribed, targeting the burning sensation that OLP patients often report [91]. Furthermore, implementing simple measures such as improved oral hygiene practices, choosing gentler toothpaste formulations, and eliminating alcohol-containing mouthwashes can significantly reduce oral discomfort. This, in turn, can lead to the alleviation of desquamative gingivitis symptoms and promote a more conducive environment for tissue healing [55,95].

To sum up, according to a recent network meta-analysis, various treatments for OLP demonstrate differing levels of efficacy and safety. Topical corticosteroids continue to be the most widely recommended treatment due to their proven effectiveness in reducing clinical symptoms and maintaining a favorable safety profile, with only minor side effects such as burning sensations and a dry mouth. Purslane emerged as a highly effective and safe treatment option, although the supporting evidence is somewhat limited. Aloe vera also showed beneficial effects with minimal adverse reactions, suggesting it could be a viable alternative for patients seeking natural remedies. In contrast, while topical calcineurin inhibitors were effective in symptom relief and clinical resolution, they were associated with a higher rate of adverse effects, including burning sensations and dysgeusia, raising concerns about their safety in long-term use [101]. Novel therapies, such as Platelet Concentrates (PCs) such as PRP and i-PRF, have shown potential in reducing pain and promoting tissue repair, especially in patients unresponsive to conventional treatments. The use of systemic therapies, immunosuppressants, retinoids, laser therapy, photodynamic therapy, vitamin D, biologics, and Janus kinase inhibitors further expands the therapeutic landscape, providing options tailored to patient-specific needs and disease severity. Surgical interventions, lifestyle modifications, and patient education also play a crucial role in the comprehensive management of OLP. The management of OLP is not only about medications and interventions [1]. The role of patient-centric measures such as education and consistent monitoring must be emphasized and patients must be informed of the potential risk of malignant transformation. Regular follow-ups involving thorough visual inspections and tactile palpation form the cornerstone of OLP monitoring [89]. Any suspicious anomalies or lesions require immediate biopsy to rule out malignancy [95].

### 3.6. Psychological Impact

It is imperative to note the psychological impact of OLP. Sorrentino et al. highlighted that psychological distress plays a significant role in the experience of patients with OLP, particularly those with erosive forms. Their multicentric study revealed that patients with keratotic OLP (K-OLP) exhibited significantly higher levels of anxiety and depression compared to healthy controls. This psychological burden can exacerbate the perception of pain and discomfort, influencing overall quality of life. Even in cases without visible lesions or pain, many OLP patients experience high levels of psychological distress, suggesting that factors such as the fear of malignant transformation and the chronic nature of the disease contribute to these mental health challenges. The study found a strong association between mood disorders and OLP, with anxiety and depression potentially serving both as triggers for the onset and exacerbation of OLP and as consequences of living with a chronic oral condition. These findings underscore the importance of incorporating psychological assessment and support into the management of OLP to address the comprehensive needs of these patients [102]. Meanwhile, Arduino et al. reported that patients with OLP often experience significant psychological distress, which can substantially impact their quality of life. Their multicenter case-control study found a high prevalence of anxiety, depression, and sleep disturbances among OLP patients compared to healthy controls. These psychological comorbidities were particularly pronounced in patients with non-keratotic OLP (nK-OLP), who reported more frequent and severe pain, often described as burning sensations that significantly impair daily activities such as eating and speaking. The unpredictable nature of OLP, coupled with chronic pain and the potential for malignant transformation, contributes to heightened emotional distress, potentially exacerbating the disease’s symptoms and progression. Additionally, mood disorders such as anxiety and depression may not only worsen the perception of pain but also impact a patient’s ability to cope with their condition, suggesting a need for comprehensive management strategies that address both the physical and psychological aspects of OLP [103].

### 3.7. Limitations and Future Directions

Investigating the long-term outcomes and prognosis of OLP patients would be valuable in guiding future research directions. Longitudinal studies assessing disease progression, potential malignant transformation, and the impact of OLP on patients’ quality of life would provide crucial insights for clinicians and patients alike. Additionally, this article could benefit from a more extensive analysis of diagnostic approaches. While it touches upon diagnostic methods such as clinical examination and histopathological evaluation, it should address potential challenges and advancements in diagnostic techniques such as molecular markers or imaging modalities. Further exploration of these diagnostic tools would aid in accurate and timely OLP diagnosis. Moreover, conducting multicenter, randomized controlled trials with larger sample sizes would help to establish evidence-based treatment guidelines and optimize therapeutic approaches.

## 4. Conclusions

The etiology of OLP, a chronic inflammatory disorder affecting the oral mucosa, is still unknown. Immunological reactions, possible changes in oral microbiota, and hormonal changes, particularly in postmenopausal women, all play a role in the development of OLP. The condition shows symptoms that range from physical symptoms such as discomfort and burning sensations to psychological ones such as reduced quality of life. According to WHO and AAOMP guidelines, diagnosis entails visual inspections for Wickham’s striae and an array of tests including cytology and biopsy. While corticosteroids continue to be the primary form of therapy, there are other modalities such as immunosuppressants, retinoids, and curcumin, as well as cutting-edge procedures such as laser therapy. Importantly, OLP has a risk of developing into OSCC, with risks determined by age, gender, and lifestyle. Although dysplasia evaluations are frequently used for risk assessment, novel biomarkers may provide more accuracy. Regular cancer screenings, biopsies, and integrated healthcare approaches are essential for optimal therapy due to the low malignancy risk associated with OLP and the adverse consequences of long-term corticosteroid usage.

## Figures and Tables

**Table 1:** Summary of the diagnostic criteria from the Modified WHO of OLP and Oral Lichenoid Lesions (2003), as well as from the American Academy of Oral and Maxillofacial Pathology 2016 [1].

Clinical Criteria	Histopathologic Criteria
Modified WHO diagnostic criteria of OLP and Oral Lichenoid Lesions (2003)	
Bilateral, more or less symmetrical lesions Reticular pattern of a lace-like network of slightly raised gray-white lines. Erosive-, atrophic-, bulbous-, and plaque-type lesions just in the presence of reticular lesions elsewhere in the oral mucosa. In all other lesions that resemble OLP but do not complete the criteria, the term “clinically compatible with” should be used [62].	Cellular infiltration (mainly lymphocytes) as a well-defined band-like zone that is confined to the superficial part of the connective tissue. Basal cell layer with signs of liquefaction degeneration. Absence of epithelial dysplasia. When the histopathological features are less obvious, the term “histopathologically compatible with” is applied [62].
Diagnostic criteria by the American Academy of Oral and Maxillofacial Pathology, 2016 [1]:	
**Multifocal Symmetric distribution** White and red lesions exhibit one or more forms: reticular/papular, atrophic (erythematous), erosive (ulcerative), plaque, and bullous. **Lesions are not localized** [63]. To the sites of smokeless tobacco placement. Adjacent to and in contact with dental restorations. **The onset of the lesion does not correlate with** [64]. The start of a medication. With the use of cinnamon-containing products.	Band-like or patchy, predominantly lymphocytic infiltrate in the lamina propria confined to the epithelium–lamina propria interface. Basal cell liquefactive (hydropic) degeneration. Lymphocytic exocytosis. Absence of epithelial dysplasia. Absence of verrucous epithelial architectural change [65].

**Table 2:** Differentiation of OLP from OLL.

Criteria	Oral Lichen Planus (OLP)	Oral Lichenoid Lesions (OLL)
Etiology and Pathogenesis	Chronic mucocutaneous disease with possible autoimmune-related etiology.	Lesions with different etiologies, such as systemic medication, dental restorative materials, or food allergens.
Clinical Presentation	Typically bilateral and symmetrical lesions, often affecting the posterior buccal mucosa, tongue, and gingiva.	More commonly unilateral lesions that may be in close contact with dental restorations or other causative agents.
Histopathological Features	Well-defined band-like inflammatory infiltrate predominantly composed of lymphocytes, saw-toothed rete ridges, and hydropic degeneration of the basal cell layer.	Infiltrates with a poorly defined lower border in the subepithelial zone and presence of acute inflammatory cells such as eosinophils and neutrophils, deeper connective tissue infiltration, and hyperkeratosis.
Response to Removal of Trigger	No improvement with removal of external factors due to idiopathic nature.	Lesions often improve or resolve upon removal or modification of the causative factor (e.g., dental restoration or medication).
Diagnosis	Diagnosis based on clinical presentation and histopathological findings, confirmed by strict band-like infiltration and atrophic epithelium.	Diagnosis involves clinical presentation, history of potential triggers, histopathological examination, and presence of deep connective tissue infiltration and hyperparakeratosis [63].

## Data Availability

Data and materials referenced in this review were derived from previously published studies in the literature. All included studies are listed in the References section.

## Abbreviations

OLP	Oral Lichen Planus
WHO	World Health Organization
OSCC	Oral Squamous Cell Carcinoma
LP	Lichen Planus
VDR	Vitamin D Receptor
EOLP	Erosive Oral Lichen Planus
SDHB	Succinate Dehydrogenase Enzyme B
CB	Cathepsin B
EADV	European Academy of Dermatology and Venereology
PDT	Photodynamic Therapy
NB-UVB	Narrowband Ultraviolet B Therapy
HLA	Human Leukocyte Antigen
IL	Interleukin
TNF	Tumor Necrosis Factor
JAK	Janus Kinase
NSAID	Nonsteroidal Anti-Inflammatory Drug
ACE	Angiotensin-Converting Enzyme
MMP	Matrix Metalloproteinase
Th	T helper cells
Tc	T cytotoxic cells
iNOS	Inducible Nitric Oxide Synthase
HCV	Hepatitis C Virus
DIF	Direct Immunofluorescence
CBC	Complete Blood Count
Hb	Hemoglobin
LRT	Lichenoid Reactions to Drugs
RNA	Ribonucleic Acid
K-OLP	Keratotic Oral Lichen Planus
nK-OLP	Non-Keratotic Oral Lichen Planus
AML	Amlexanox
AV	Aloe Vera
CUR	Curcumin
PLA	Placebo
PUR	Purslane
SysCORT	Systemic Corticosteroid
TopCALN	Topical Calcineurin
TopCALNcoSysCORT	Topical Calcineurin combined with Systemic Corticosteroid
TopCORT	Topical Corticosteroid
